# Advances in Cancer Treatment by Targeting the Neddylation Pathway

**DOI:** 10.3389/fcell.2021.653882

**Published:** 2021-04-08

**Authors:** Wenbin Gai, Zhiqiang Peng, Cui Hua Liu, Lingqiang Zhang, Hong Jiang

**Affiliations:** ^1^Department of Physiology, Shandong Provincial Key Laboratory of Pathogenesis and Prevention of Neurological Disorders and State Key Disciplines: Physiology, School of Basic Medicine, Medical College, Qingdao University, Qingdao, China; ^2^State Key Laboratory of Proteomics, Beijing Proteome Research Center, National Center for Protein Sciences (Beijing), Beijing Institute of Lifeomics, Beijing, China; ^3^CAS Key Laboratory of Pathogenic Microbiology and Immunology, Institute of Microbiology, Chinese Academy of Sciences, Beijing, China; ^4^Peixian People’s Hospital, Xuzhou, China

**Keywords:** developmental down-regulation protein 8 (NEDD8), neddylation, MLN4924, treatment, cancer

## Abstract

Developmental down-regulation protein 8 (NEDD8), expressed by neural progenitors, is a ubiquitin-like protein that conjugates to and regulates the biological function of its substrates. The main target of NEDD8 is cullin-RING E3 ligases. Upregulation of the neddylation pathway is closely associated with the progression of various tumors, and MLN4924, which inhibits NEDD8-activating enzyme (NAE), is a promising new antitumor compound for combination therapy. Here, we summarize the latest progress in anticancer strategies targeting the neddylation pathway and their combined applications, providing a theoretical reference for developing antitumor drugs and combination therapies.

## Introduction

As a post-translational modification, protein neddylation refers to a process where substrate proteins are tagged with a ubiquitin-like protein NEDD8 and participate in cellular activity by regulating protein function. NEDD8 encodes an 81-amino acid polypeptide, which is highly homologous to ubiquitins and is connected to its substrates by forming isopeptide chains. For NEDD8, this linkage occurs between Gly-76 at NEDD8’s C-terminus and the Lys-48 residue of the substrates (Kamitani et al., 1997). Different from ubiquitin, as a precursor, NEDD8 is initially synthesized with five additional downstream residues of Gly-76 that must be cracked by a C-terminal hydrolase (Rabut and Peter, 2008), mainly ubiquitin carboxyl-terminal esterase L3 (UCH-L3) (Johnston et al., 1997) and NEDD8 specific-protease cysteine (NEDP1) (Gan-Erdene et al., 2003; Mendoza et al., 2003). After that, an adenosine triphosphate (ATP) and an E1 NEDD8-activating enzyme (NAE) first adenylate and activate mature NEDD8, respectively. NAE is a heterodimer comprising NAE1 (also called APPBP1) and UBA3 (also called NAEβ) (Bohnsack and Haas, 2003; Walden et al., 2003; Kurz et al., 2008). Next, activated NEDD8 transfers to one of two NEDD8-conjugating E2 enzymes (UBC12/UBE2M or UBE2F) (Kamitani et al., 1997; Huang et al., 2005). Finally, the E3 ligase catalyzes the production of isomers of the C-terminal Gly-76 and lysine residue of the substrate protein via covalent attachment, ultimately transferring NEDD8 to the substrates to complete the neddylation process (Kamitani et al., 1997).

E3 ubiquitin ligases are numerous, but 10 NEDD8 E3 ligases are available. Except for defective cullin neddylation 1 (DCN1) (Kurz et al., 2005, 2008) and DCN1-like proteins (Kurz et al., 2008; Meyer-Schaller et al., 2009), most of these contain the novel gene (RING) domain structure. The 10 NEDD8 E3 ligases are DCN1, RING-box proteins 1 (RBX1) and RBX2 [also known as regulators of cullin 1 (ROC1) and ROC2/SAG, respectively] (Duan et al., 1999; Kamura et al., 1999; Huang et al., 2009), murine double minute 2 (MDM2) (Xirodimas et al., 2004), casitas B-lineage lymphoma (c-CBL) (Oved et al., 2006; Zuo et al., 2013), SCFFBXO11 (Zuo et al., 2013), ring finger protein 111 (RNF111) (Ma et al., 2013), inhibitors of apoptosis (IAPs) (Broemer et al., 2010), TFB3 (TFIIH/NER complex subunit TFB3) (Rabut et al., 2011), and tripartite motif containing 40 (TRIM40) (Noguchi et al., 2011). The RING-type neddylation ligase acts as a scaffold to bind the E2 ubiquitin complex directly to the substrate, enhancing ubiquitin transfer to the substrate protein (Metzger et al., 2014). Different from RING-type neddylation ligases, HECT-type neddylation ligases act catalytically by constituting a thioester bond with the C-terminal lobe of the HECT domain before the transfer of ubiquitin to its intended substrate (Berndsen and Wolberger, 2014; Zheng and Shabek, 2017). HECT-type neddylation ligases remain less defined than RING-type neddylation ligases, such as Yeast Rsp5, Itch (Li et al., 2016) (E3 ubiquitin-protein ligase Itchy homolog), Smad ubiquitination regulatory factor 1 (Smurf1) (Xie et al., 2014), Smad ubiquitination regulatory factor 2 (Smurf2) (Shu et al., 2016), NEDL1 (NEDD4-like E3 ubiquitin-protein ligase 1) and NEDL2 (NEDD4-like E3 ubiquitin-protein ligase 1) (Qiu et al., 2016) (Table 1). Furthermore, all NEDD8 E3 ligases identified thus far can be used as ubiquitin E3 ligases (Zhao et al., 2014).

**TABLE 1:** Classification of NEDD8 E3 ligases.

**Neddylation E3 ligases**		
**HECT E3s**	**RING E3s**	
Itch NEDL1 NEDL2 Smurf1 Smurf2 Yeast Rsp5	CBLs DCN1 IAPs MDM2 RNF111 Roc1/2	SCFFBXO11 TFB3 TRIM40

**FIGURE 1:**
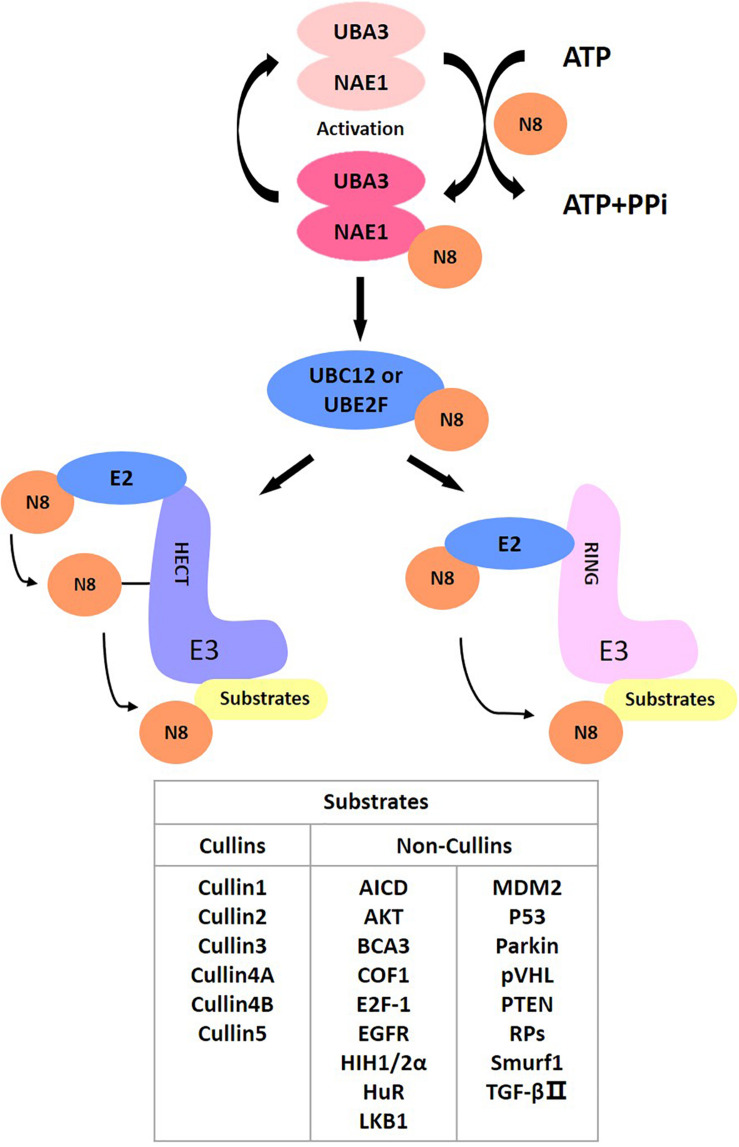
The process of neddylation modification of proteins.

NEDD8 regulates the activities of substrates and participates in various signaling pathways, including cell proliferation, autophagy and transformation. Cullins are the most typical target proteins for neddylation. Typical substrates of cullin-RING ligases (CRLs) include proteins related to cell cycle regulation (e.g., Cyclin D/E, p21, p27, and WEE1) (Jia et al., 2011; Luo et al., 2012; Gao et al., 2014; Li et al., 2014; Hua et al., 2015; Paiva et al., 2015; Han et al., 2016; Lan et al., 2016; Xie et al., 2017; Zhang et al., 2016), apoptosis (e.g., BIM, NOXA, BIK, Bcl-xL, Mcl-1, and c-FLIP) (Jia et al., 2011; Dengler et al., 2014; Godbersen et al., 2014; Yao et al., 2014; Knorr et al., 2015; Chen et al., 2016; Czuczman et al., 2016; Leclerc et al., 2016; Tong et al., 2017; Wang et al., 2017) and signal transduction pathways (e.g., HIF1α, REDD1, β-catenin, and Deptor) (Milhollen et al., 2010; Swords et al., 2010; Zhao et al., 2012; Godbersen et al., 2014). Activation of CRLs contributes to cancer progression and degradation of their substrates (Xirodimas, 2008). In addition to cullins, several other targets of neddylation, involving tumor suppressor p53 (Xirodimas et al., 2004), Hu antigen R (HuR) (Stickle et al., 2004), von Hippel-Lindau protein (pVHL) (Stickle et al., 2004; Embade et al., 2012), epidermal growth factor receptor (EGFR) (Oved et al., 2006), oncoprotein mouse double minute 2 (Mdm2) (Xirodimas et al., 2004), ribosomal proteins (Xirodimas et al., 2008), AKT, liver kinase B1 (LKB1) (Barbier-Torres et al., 2015), and PTEN (Xie et al., 2020), also effectively affect disease onset and progression. Therefore, targeting neddylation is an effective treatment for treating disease (Figure 1).

The substrate properties dictate the critical effect of neddylation in regulating biological processes and disease management. Recent studies have proposed the relevance of neddylation modifications in cell cycle control, DNA replication regulation, cell cycle progression and cell division. The neddylation pathway is hyperactivated during human cancer evolution (Zhou L. et al., 2019). Blocking the neddylation pathway has become an appealing anti-cancer treatment (Jiang and Jia, 2015). However, inhibiting the neddylation pathway significantly upregulates the expression of the T-cell minus modulator programmed death-ligand 1 (PD-L1), possibly explaining the underlying resistance by evading immune surveillance checkpoints (Zhou S. et al., 2019). In this review, we summarize and analyse the promising potential of the targeted neddylation pathway as a new therapeutic method and effects of MLN4924/pevonedistat/TAK-924 treatment combined with other anticancer therapies, particularly those targeting the antitumor immune axis.

## Target Proteins of Neddylation

After activation by neddylation, CRLs are the largest group of multi-unit E3 ubiquitin ligases responsible for ubiquitination, with roughly 20 percent of cellular proteins targeted then degraded by the ubiquitin-proteasome system (UPS) (Petroski and Deshaies, 2005). The connection between NEDD8 and the lysine residues at the C-terminus of cullins activates CRLs (Sakata et al., 2007; Merlet et al., 2009), resulting in a structural alteration in the CRL complex: it adopts an open conformation to increase the entry of ubiquitinated substrates (Zheng et al., 2002; Duda et al., 2008; Saha and Deshaies, 2008). CRL is a multi-unit E3 comprising the following four components: cullin, a substrate recognition receptor, an adaptor protein, and one RING protein. There are eight cullins, including CUL1-3, CUL4A, CUL4B, CUL5, CUL7, and CUL9, which are the optimal substrates of the NEDD8 pathway; they have an evolutionarily conserved cullin homology domain (Petroski and Deshaies, 2005). Every cullin protein is regarded as a molecular framework that promotes the combination of an adaptor protein, an N-terminal substrate receptor protein and a C-terminal RING protein (RBX1 or RBX2) to assemble a CRL (Feldman et al., 1997; Deshaies, 1999; Seol et al., 1999; Petroski and Deshaies, 2005). CRLs regulate many important biological processes, such as cell survival, apoptosis, genomic integrity, tumourigenesis and signal transduction, by facilitating the ubiquitination and degradation of critical zymolytes (Feldman et al., 1997; Nakayama and Nakayama, 2006; Deshaies and Joazeiro, 2009).

Cullin neddylation activates CRLs, but some non-cullin proteins are also protein substrates of neddylation. In 1979, p53 was originally recognized as a factor related to transformation, and researchers have gradually discovered that it is closely associated with the tumor process. *In vivo*, p53 modifications occur mainly in pathways that promote ubiquitination, phosphorylation and acetylation (Brooks and Gu, 2003). Research has indicated that p53 is an essential target for neddylation as well. The stability and function of p53, a tumor suppressor, are tightly regulated by post-translational modifications, including ubiquitylation and neddylation, in which the MDM2 oncoprotein plays a critical role. Mdm2, as an E3 ubiquitin ligase, binds directly to p53, thereby promoting its polyubiquitination and proteasomal degradation (Nakamura et al., 2000). Furthermore, Mdm2 and F-box protein 11 (FBXO11) facilitate the combination of NEDD8 with p53, thus inhibiting p53 activity (Xirodimas et al., 2004; Abida et al., 2007). Several ribosomal proteins have been identified as potential NEDD8 substrates (Xirodimas et al., 2008). L11 was found to be neddylated by Mdm2 and deneddylated by NEDP1. MDM2-mediated L11 neddylation protects L11 from degradation, and both L11 (Lohrum et al., 2003; Zhang et al., 2003) and S14 (Zhou et al., 2013) bind to MDM2 and regulate p53 stability. Furthermore, the expression level of the RNA-binding protein HuR is associated with MDM2. HuR could be protected from degradation by neddylation through Mdm2-dependent stabilization (Embade et al., 2012). Other non-cullin substrates of neddylation have been reported, including the following: the tumor suppressor pVHL (Stickle et al., 2004); receptor proteins such as EGFR (Oved et al., 2006) and TGF-β type II receptor (Zuo et al., 2013); and transcriptional regulators such as HIF1α/HIF2α (Ryu et al., 2011), breast cancer-associated protein 3 (BCA3) (Gao et al., 2006), APP intracellular domain (AICD) (Lee et al., 2008), E2F-1 (Loftus et al., 2012), HECT-domain ubiquitin E3 ligase SMURF1 and RBR ubiquitin E3 ligase Parkin (Xie et al., 2014; Enchev et al., 2015). Additionally, new potential neddylation targets exist for LKB1 and Akt (Barbier-Torres et al., 2015).

In addition to the substrates mentioned above, Vogl et al. (2020) recently developed a series of NEDD8-ubiquitin-substrate spectra (sNUSP) that can be used to identify new substrates, such as COF1. The identification of a growing number of substrates suggests that neddylation plays an extensive role in cells with more complex cancer-promoting mechanisms than previously thought, providing a theoretical basis for targeting the neddylation pathway in the treatment of various diseases.

## Targeting Protein Neddylation as an Anticancer Strategy

NEDD8 was initially identified as a gene whose expression is downregulated during development in the mouse brain (Kumar et al., 1992). However, it was demonstrated subsequently to exist in various mouse tissues and is highly conserved in vertebrate species and somewhat conserved in yeast (Kumar et al., 1993), suggesting that the neddylation pathway is essential during species evolution. Neddylation is a type of posttranslational modification that modulates substrate protein activity. Neddylation modification is catalyzed by an NAE (E1), a NEDD8-conjugating enzyme (E2), and a NEDD8 ligase (E3); these factors link a ubiquitin-like molecule, NEDD8, to the lysine residues of the substrate protein. Accumulating evidence shows that NEDD8 is overexpressed in some human diseases, such as neurodegenerative disorders (Dil Kuazi et al., 2003; Mori et al., 2005) and cancers (Chairatvit and Ngamkitidechakul, 2007; Salon et al., 2007). Thus, targeting protein neddylation has recently been recognized as a popular anticancer method (Watson et al., 2011; Zhou et al., 2018). We summarized previous and recent findings in Table 2.

**TABLE 2:** Neddylation modification as an inhibition.

**Ligase**	**Product Name**	**Mechanism and Principal Action**	**Target**	**References**
E1	MLN4924	Pevonedistat (MLN4924) inhibits NAE activity more selectively than the closely related ubiquitin-activating enzyme (UAE, also known as UBA1) and SUMO-activating enzyme (SAE; a heterodimer of SAE1 and UBA2 subunits), in purified enzyme and cellular assays. MLN4924 exhibits potent cytotoxic activity against a variety of human tumor-derived cell lines.	NAE1	Soucy et al., 2009
E2	WS-383	WS-383 is a potent, selective and reversible inhibitor of the DCN1-UBC12 interaction. WS-383 inhibits Cul3/1 neddylation and induces the accumulation of p21, p27 and NRF2.	DCN1-UBC12 interaction	Wang et al., 2019
	DI-591	DI-591 binds to purified recombinant human DCN1 and DCN2 protein and disrupts the DCN1-UBC12 interaction in cells. Treatment with DI-591 selectively converts cellular cullin 3 into an un-neddylated inactive form with no or minimum effect on other cullin members.	DCN1-UBC12 interaction	Zhou H. et al., 2017
	NAcM-OPT	NAcM-OPT is an orally bioavailable cullin neddylation 1 (DCN1) inhibitor, which potently inhibits the DCN1-UBE2M interaction.	DCN1	Hammill et al., 2018

### NEDD8-Activating Enzyme (NAE)

NEDD8 is activated through an ATP-dependent reaction via NAE and then is transferred to NEDD8-conjugating enzyme E2. MLN4924 is a selective, effective, first-rate inhibitor of NAE (Gong and Yeh, 1999). This micromolecule inhibits the protein neddylation pathway and is currently under multiple clinical investigations of its anticancer effect against solid tumors and leukemia (Soucy et al., 2009; Godbersen et al., 2014; Swords et al., 2018). The MLN4924 antitumor activity is mediated by its ability to induce cell-associated autophagy, apoptosis and senescence (Milhollen et al., 2010; Han et al., 2016). For example, in liver cancer, MLN4924 induces the DNA damage response (DDR) and apoptosis to inhibit hepatoma cell development *in vitro* and *in vivo* and also induces autophagy, whereas MLN4924 induces autophagy mediated by accumulating the mTOR inhibitory protein Deptor and inducing reactive oxygen species (ROS)-mediated oxidative stress (Peterson et al., 2009; Luo et al., 2012). Identical to its effect in liver cancer, MLN4924 effectively suppresses lymphoma cell growth by inducing cycle arrest of G2 cells and subsequent cell line-dependent apoptosis or senescence. Apoptosis induced by MLN4924 is mediated by the apoptotic signaling pathway, with significantly upregulated pro-apoptotic proteins Bik and Noxa and downregulated anti-apoptotic proteins XIAP, c-IAP1 and c-IAP2, while aging induced by neddylation suppression seemingly depends on the expression of tumor suppressors p21/p27 (Brownell et al., 2010). Mechanistically, when tumor cells are treated with MLN4924, MLN4924 blocks the activities of NAE by binding to its active site to constitute a covalent NEDD8-MLN4924 adduct. Therefore, CRLs are inactivated, leading to the accumulation of tumor-suppressive substrates of CRLs and apoptosis or senescence induction to inhibit cancer cell progression (Karin et al., 2002; Wang et al., 2015).

Consistent with NAE inhibition, MLN4924 treatment of cultured tumor cells results in the inhibition of CRL neddylation and a reciprocal rise in the levels of foregone CRL substrates such as p-IκBα (Soucy et al., 2009). The accumulation of p-IκBα in the cytoplasm inhibits the nuclear translocation of NF-κB transcription factors and suppresses the NF-κB pathway, affecting tumourigenesis and development through transcriptionally controlling genes related to cell growth, angiogenesis, apoptosis, metastasis and cell migration (Karin et al., 2002). For example, in activated B-cell-like diffuse large B-cell lymphoma (ABC-DLBCL), MLN4924 causes G1-phase cell cycle arrest and apoptosis induction by blocking the classic NF-κB pathway. Thus, MLN4924 treatment leads to G1 phase arrest, P-IκBα accumulation and decreased inhibition of NF-κB target genes, significantly affecting MLN4924-mediated antitumor effects (Milhollen et al., 2010).

Autophagy plays a critical role in maintaining cellular homeostasis and is closely associated with the development of many human diseases (Wang and Zhang, 2019). MLN4924 significantly inhibits CRL neddylation modifications and effectively induces autophagy in both dose- and time-dependently in multiple human cancer cell lines (Zhao et al., 2012). MLN4924 inhibits the activity of CRLs, induces the accumulation of its substrate IκBα, blocks the activation of NF-κB and expression of catalase, and promotes the expression of ATF3, thereby inducing autophagy in oesophageal cancer cells (Liang et al., 2020). mTOR is a well-established negative regulator of autophagy (Kim and Guan, 2015). By inactivating CRLs/SCF E3s, MLN4924 can inhibit mTORC1 activity by causing DEPTOR accumulation directly and DEPTOR and HIF1α accumulation via the HIF1-REDD1-TSC1 axis (HIF1α) (Zhao et al., 2012). MLN4924 also triggers autophagy in colon cancer cells by suppressing the PI3K/AKT/mTOR pathway (Lv et al., 2018). Autophagy may be a novel anti-cancer mechanism for MLN4924 in cancer treatment, providing conceptual evidence for the strategic combination of MLN4924 with autophagy inhibitors to maximize tumor cell killing through enhanced apoptosis.

MLN4924 leads to DNA re-replication, which triggers checkpoint activation, apoptosis, and senescence in cancer cells (Soucy et al., 2009). The replication of genetic material is a critical process of the cell cycle. Re-replication is a known signal that induces DNA damage and causes DNA damage signaling in cells (Zhu et al., 2004; Archambault et al., 2005). Cdt1 is the initiation factor for the induction of DNA re-replication in cells treated with MLN4924 (Lin et al., 2010). Similarly, the DNA damage signaling factors P21 and P53 are important substrates of the NEDD8-mediated neddylation pathway. P21 is crucial in the S-phase of the cell cycle, DNA replication and the cellular senescence pathway (Pérez-Yépez et al., 2018). MLN4924-induced senescence in human colorectal cancer cells relies on recruiting p53 and its downstream adaptor P21 (Lin et al., 2010). For other human tumor-derived cell lines, including HCT116 (colon), Calu-6 (lung), SKOV-3 (ovarian), H460 (lung), DLD-1 (colon), MCF-7 (mammary gland), CWR22 (prostate) and OCI-LY19 (lymphoma), MLN4924 treatment also inhibits proliferation and migration.

Currently, phase I trials for MLN4924 are ongoing in cancers, such as metastatic melanoma (Bhatia et al., 2016), advanced solid tumors (Bhatia et al., 2016), acute myeloid leukemia (Swords et al., 2015), myelodysplastic syndromes (Shah et al., 2016), lymphoma and multiple myeloma (Shah et al., 2016), and these studies have revealed that critical therapeutic effects can be obtained by antagonizing NEDD8-mediated protein degradation (Supplementary Table 1).

Although excellent activity of MLN4924 was observed in early trials, drug resistance was also found in large number of patients. Early preclinical studies have shown that treatment-emergent NAEβ mutations promotes resistance to MLN4924. Additionally, in human leukemic cells, UBA3 mutations increase the enzyme’s affinity for ATP while decreasing its affinity for NEDD8 (Milhollen et al., 2012; Xu et al., 2014); these mutations effectively contribute to decreased MLN4924 potency *in vitro*. In TCGA, PanCancer Atlas, the frequency of mutations in UBA3 is about 20%, that may suggest that mutations in UBA3 are not the main cause of MLN4924 resistance. Mutations of key molecules are often associated with drug resistance, and in addition to mutations of NAEβ and UBA3, the upregulation of ABCG2 transcription in resistant cells drives clinical resistance (Kathawala et al., 2020; Wei et al., 2020). Thus, MLN4924 is widely used as an anti-cancer drug in clinical practice but still has some limitations.

### NEDD8-Conjugating Enzyme

Activated NEDD8 can be transferred to the subunits of the substrate by the NEDD8-conjugating enzyme E2, which includes two members: UBE2F and UBE2M/Ubc12. RBX proteins can be divided into RBX1 and RBX2 in humans (Nakamura et al., 2000; Brooks and Gu, 2003; Nakayama and Nakayama, 2006; Abida et al., 2007). UBE2F pairs with RBX2 to modulate cullin 5 neddylation dependent on E2 RING, while UBE2M functions through RRB1 to mediate the neddylation of cullin 1, 2, 3, 4a, 4b, and 7 (Zhou W. et al., 2017). The E2-RBX-cullin interaction combination determines the *in vivo* selectivity of neddylation (Huang et al., 2009). The cellular levels of different RBX partners determine the cellular levels of distinct cullins. The two NEDD8 E2s exert different effects in cullin neddylation *in vivo* (Huang et al., 2009).

Inhibition of E2s, which inhibit one subset of NEDD8 substrates compared with all neddylation substrates, may provide better cytotoxic selectivity than inhibition of E1s, which inactivates the entire neddylation pathway. In lung cancer, targeting UBC12 causes accumulation of the CRL substrates p21, p27, and Wee1, inactivating CRL ubiquitin ligase and arresting the cell cycle in the G2 phase (Li et al., 2019). Therefore, targeting E2s to inhibit neddylation modification blocks the protein neddylation pathway and deactivates CRLs, triggering the aggregation of tumor-suppressive CRL substrates, stopping the cell cycle and impeding tumor growth and metastasis.

### NEDD8 E3 Ligases

E3 ubiquitin ligases based on Cullin are activated by NEDD8 binding to Cullins. Therefore, targeting E2s to inhibit neddylation modification blocks the protein neddylation pathway and deactivates CRLs, triggering the aggregation of tumor-suppressive CRL substrates, stopping the cell cycle and impeding tumor growth and metastasis (Kurz et al., 2008). Human cells express 5 DCN1-like (DCNL) proteins, termed DCNL1–DCNL5 (also named DCUN1D1–5), each encompassing a C-terminal potentiating neddylation domain and an N-terminal ubiquitin-binding (UBA) domain, which we termed the PONY domain, with distinct amino-terminal extensions (Kurz et al., 2005; Kurz et al., 2008; Meyer-Schaller et al., 2009). For example, in various human tumors, activation of squamous cell carcinoma-associated oncogene (SCCRO) triggers its function as an oncogene, and the UBA domain in SCCRO (also called DCUN1D1) works as a feedback regulator of biochemical and oncogenic activity (Huang et al., 2015). Conversely, DCNL3 levels are downregulated in the liver, bladder, and renal tumors (Ma et al., 2008) compared with those in normal controls, indicating that DCNL regulation is critical for human cancer development. Considering the conserved binding characteristics of the UBA domain, targeting these vital proteins could possess therapeutic implications for human cancer treatment.

## Targeting Protein Neddylation-Based Combination Therapies

### NAE Inhibitor MLN4924 Combined With Chemotherapy Drugs

The effectiveness of radiotherapy for cancer is limited by some of the toxic side effects of dose increases, although existing radiotherapy remains the preferred problem for local cancer control (Lyons et al., 2011; Venur and Leone, 2016). Chemotherapy can improve the efficiency of ionizing radiation by inhibiting DNA repair and overcoming apoptotic resistance (Bandugula and N, 2013). Among anticancer drugs, 2-deoxy-D-glucose (2-DG) is the most effective inhibitor of glycolysis, glucose metabolism and ATP production (Dwarakanath, 2009). 2-DG increases the efficacy of chemotherapy drugs (such as doxorubicin [DOX] and paclitaxel) in human osteosarcoma and non-small cell lung cancer *in vivo* (Kern and Norton, 1987). 2-DG + DOX and buthionine sulfoximine (BSO) dramatically promotes cytotoxicity by regulating oxidative stress and interfering with thioethanol metabolism in breast cancer cells (Tagg et al., 2008). MLN4924 can sensitize drug-resistant pancreatic, lung and breast cancer cells to ionizing radiation, although it has little effect on normal lung fibroblasts, indicating that MLN4924 is a new radiation sensitizer (Wei et al., 2012; Yang et al., 2012; Sun and Li, 2013). Therefore, 2-DG plus MLN4924 could be an anti-proliferative and radiation-sensitizing strategy for various human cancers, providing insights on breast cancer treatment (Oladghaffari et al., 2017).

### NAE Inhibitor MLN4924 Combined With Targeted Drugs

Endocrine therapy is the standard treatment for oestrogen receptor (ER)-positive breast cancer and can significantly reduce the risk of disease recurrence and mortality (Anbalagan and Rowan, 2015). However, nearly one-third of patients still experience disease recurrence and metastasis mediated by endocrine resistance at the beginning of treatment or during treatment (deConinck et al., 1995; Anbalagan and Rowan, 2015). Fulvestrant has been approved as a selective oestrogen receptor downregulator (SERD) to cure locally advanced or metastatic breast carcinoma and significantly extends the progression-free survival of patients (Johnston and Cheung, 2010). The neddylation modification pathway is activated in breast carcinoma and is associated with ER-α expression. In anti-breast cancer treatment, the neddylation pathway can downregulate ER-α expression and inhibit ER inactivation, which can have a synergistic anticancer effect with fulvestrant (Jia et al., 2019).

Inhibitors of apoptosis proteins (IAPs) are anti-apoptotic regulators that prevent apoptosis and are often overexpressed in many human tumors, in which they promote apoptosis evasion and cell survival (Gyrd-Hansen and Meier, 2010). IAP antagonists, also regarded as second mitochondria-derived activator of caspase (SMAC) mimetics, have been recognized as new apoptosis-inducing agents for treatment, either alone or in combination with other antitumor drugs (Dineen et al., 2010; Sumi et al., 2013). MLN4924 activates stress-response signaling and works synergistically with IAP antagonists and DNA damage-inducing chemotherapies. The oral IAP antagonist T-3256336 synergistically promotes the anti-proliferative results of the NAE inhibitor MLN4924 in cancer cells (Sumi et al., 2016). The combination of IAP antagonists with MLN4924 inhibits tumor proliferation, demonstrating the promise of a novel cancer combination treatment.

### NAE Inhibitor MLN4924 Combined With Drugs Targeting the Antitumor Immune Axis

Because the FDA approved the anti-PD-1 (programmed death-1) antibodies nivolumab and pembrolizumab, as well as the anti-PD-L1 antibodies atezolimuab, durvalumab and avelumab, the signaling pathway involving PD-1 and its ligand PD-L1 has become a research hotspot in the field of tumor immunology and oncology (Dong et al., 2002). However, not all tumors are sensitive to these compounds. Inhibitors of neddylation are potential cancer treatment and may promote cancer-related immunosuppression. Increasing evidence has demonstrated that some traditional and targeted cancer therapies modulate antitumor immunity (Galluzzi et al., 2015; Patel and Minn, 2018), suggesting that cytotoxic anticancer drugs combined with immune checkpoint blockade therapy may be an effective combination. Thus, the combination of MLN4924 and anti-PD-L1 therapy might significantly increase the therapeutic efficacy *in vivo* compared with that with either agent alone.

## Conclusion

MLN4924/pevonedistat/TAK-924, as a micromolecule inhibitor, inhibits NEDD8-activating enzyme (NAE), which impedes the ubiquitination modification cascade, inactivating CRLs. MLN4924 is the critical element of the dynamic protein homeostasis pathway. Many clinical studies have shown the impressive antitumor activity of MLN4924, but single-drug treatment has some limitations. Clinical trials have demonstrated that MLN4924 alone or combined with chemotherapy has a good treatment effect. MLN4924 is currently under phase II/III clinical trials for antitumor treatment and shows good safety and tolerability, indicating its good development prospects. We summarize the previous and recent findings in Supplementary Table 1.

Recent studies have shown that MLN4924 has good anti-ubiquitination activity and several activities independent of its ubiquitination effects. MLN4924 induces EGFR dimerization, thus triggering AKT1 activation. However, AKT1 and EGFR inhibitors can eliminate MLN4924’s inhibition of cilia formation (Mao et al., 2019). These results suggest that MLN4924 may have new applications in human cancer therapy that exhibit cilia-dependent increase or drug resistance (Zhou et al., 2016). MLN4924 can also promote glycolysis, and MLN4924 significantly increases the activity of pyruvate kinase (PK), which could improve the survival rate of breast carcinoma cells. Therefore, PKM2 activation, which promotes glycolysis and cell survival, is an adverse outcome of MLN4924 for cancer treatment and careful monitoring is required when using this drug (Zhou Q. et al., 2019). The dosage of MLN4924 is also worthy of our attention. Studies on various signal inhibitors have shown that the tumor sphere stimulation of MLN4924 is primarily regulated by the RAS/MAPK pathway. In mouse skin, MLN4924 accelerates EGF-induced injury recovery. Therefore, a low dose of MLN4924 controls the proliferation and differentiation of stem cells and has different anticancer properties than the high dose. Additionally, MLN4924 has promising application in stem cell treatment and tissue regeneration. In addition to the dosage of MLN4924 that requires caution, the drug resistance of MLN4924 also deserves our attention. In TCGA, PanCancer Atlas, the frequency of mutations in UBA3 in all tumors is approximately 20%, which may suggest that there are other reasons for MLN4924 resistance and that no key gene mutations but the upregulation of ABCG2 transcripts were found in relapsed/refractory patients with MLN4924. Therefore, we can use this hint to look for other causes of drug resistance in MLN4924, and that bring new understanding to the resistance of MLN4924 to better overcome it. To overcome resistance to MLN4924, refining the drug combination may be a more routine and convenient clinical tool, in contrast to the development of a new generation of NAE inhibitors. In parallel, mutant molecules or ABCG2 can be used as clinical biomarkers to predict therapeutic resistance to MLN4924.

Immunotherapy has become a hot topic in cancer precision medicine and has gradually developed into the fourth tumor treatment mode after surgery, chemotherapy and radiotherapy. However, it is not universally effective, and even the most popular PD-1/PDL-1 therapy only leads to a good response in approximately 20% of patients. The body’s immune system has the function of immune surveillance. When malignant cells appear in the body, the immune system recognizes and specifically clears these “non-self” cells. However, tumor cells can still grow in the body, suggesting that they can either evade attack by the host immune system or somehow modulate the body’s effective antitumor immune response. The inhibition of cell activation caused by tumor cell modification is an important mechanism of tumor immune escape. According to recent research progress, targeted therapy is expected to inhibit tumor immune escape, improve the therapeutic effect of tumor treatment and improve the prognosis of patients.

## Author Contributions

LZ, HJ, and CL contributed to the conception of the review. WG wrote the manuscript. ZP helped perform the analysis with constructive discussions. All the authors contributed to the article and approved the submitted version.

## Conflict of Interest

The authors declare that the research was conducted in the absence of any commercial or financial relationships that could be construed as a potential conflict of interest.

## Supplementary Material

Supplementary Table 1 Combined application and clinical staging of MLN4924.
