# Supplementary material for: Advances in Cancer Treatment by Targeting the Neddylation Pathway
Source: Front Cell Dev Biol. 2021 Apr 8;9:653882. doi: 10.3389/fcell.2021.653882 (PMC8060460; doi:10.3389/fcell.2021.653882)
Supplement: Supplementary Table 1 — Combined application and clinical staging of MLN4924. [file Data_Sheet_1.docx]

**Supplementary Table 1.** Combined application and clinical staging of MLN4924.

| Intervention/treatment | Tumour | Phase | Sponsor | ClinicalTrials.gov Identifier | Status | Study Results | Enrolments | Date | Reference |
| --- | --- | --- | --- | --- | --- | --- | --- | --- | --- |
| Drug: Belinostat  Drug: MLN4924 | Recurrent Acute Myeloid Leukaemia  Refractory Acute Myeloid Leukaemia | 1 | National Cancer Institute (NCI) | NCT03772925 | Recruiting | No Results | 45 | Study Start:  February 28, 2019  Study Completion:  July 1, 2021 | NO |
| Drug: MLN4924  Drug: Azacitidinet | Acute Myelogenous Leukaemia  Acute Lymphoblastic Leukaemia | 2 | Millennium Pharmaceuticals, Inc. | NCT00911066 | Completed | Complete Response | 72 | Study Start:  June 2009  Study Completion:  October 2013 | (107) |
| Drug: Decitabine  Drug: MLN4924 | Acute Myeloid Leukaemia  Recurrent Acute Myeloid Leukaemia  Refractory Acute Myeloid Leukaemia  Secondary Acute Myeloid Leukaemia  Therapy-Related Acute Myeloid Leukaemia  Untreated Adult Acute Myeloid Leukaemia | 1 | City of Hope Medical Center \| National Cancer Institute (NCI) | NCT03009240 | Recruiting | No Results | 30 | Study Start:  July 5, 2017  Study Completion:  July 5, 2021 | NO |
| Drug: MLN4924  Drug: Cytarabine | Acute Myelogenous Leukaemia  Advanced Myelodysplastic Syndromes | 1 | Justin Watts, MD \| Takeda \| University of Miami | NCT03459859 | Recruiting | No Results | 18 | Study Start:  May 21, 2018  Study Completion:  March 2023 | NO |
| Drug: MLN4924 | Advanced Nonhaematologic Malignancies | 1 | Millennium Pharmaceuticals, Inc. | NCT00677170 | Completed | Stable Disease | 62 | Study Start:  April 2008  Study Completion:  December 2012 | 35 |
| Drug: MLN4924  Drug: Docetaxel  Drug: Carboplatin  Drug: Paclitaxel | Advanced Solid Tumours | 1 | Millennium Pharmaceuticals, Inc. \| Takeda | NCT03057366 | Completed | Not Reported | 8 | Study Start:  May 11, 2017  Study Completion:  November 5, 2018 | NO |
| Drug: MLN4924  Drug: Etoposide  Drug: Prednisone  Drug: Vincristine  Drug: Cyclophosphamide  Drug: Doxorubicin  Drug: Rituximab  Drug: Filgrastim | Diffuse Large B-cell Lymphoma | 1/2 | National Cancer Institute (NCI) \| National Institutes of Health Clinical Center (CC) | NCT01415765 | Withdrawn | No Results | 0 | Study Start:  July 15, 2011  Study Completion:  January 7, 2014 | NO |
| Drug: MLN4924 | Haematologic Malignancies  Multiple Myeloma  Lymphoma  Hodgkin Lymphoma | 1 | Millennium Pharmaceuticals, Inc. | NCT00722488 | Completed | Partial Response | 56 | Study Start:  June 2008  Study Completion:  September 2013 | (108) |
| Drug: Ixazomib Citrate  Drug: MLN4924 | Recurrent Plasma Cell Myeloma  Recurrent Plasma Cell Myeloma | 1 | National Cancer Institute (NCI) | NCT03770260 | Recruiting | No Results | 66 | Study Start:  April 23, 2019  Study Completion:  April 23, 2021 | NO |
| Drug: Carboplatin  Drug: Paclitaxel  Drug:MLN4924 | Metastatic Cholangiocarcinoma  Metastatic Hepatocellular Carcinoma  Stage III Hepatocellular Carcinoma AJCC v8  Stage III Intrahepatic Cholangiocarcinoma AJCC v8  Stage IIIA Hepatocellular Carcinoma AJCC v8  Stage IIIA Intrahepatic Cholangiocarcinoma AJCC v8  Stage IIIB Hepatocellular Carcinoma AJCC v8  Stage IIIB Intrahepatic Cholangiocarcinoma AJCC v8  Stage IV Hepatocellular Carcinoma AJCC v8  Stage IV Intrahepatic Cholangiocarcinoma AJCC v8  Stage IVA Hepatocellular Carcinoma AJCC v8  Stage IVB Hepatocellular Carcinoma AJCC v8  Unresectable Cholangiocarcinoma  Unresectable Hepatocellular Carcinoma  Unresectable Intrahepatic Cholangiocarcinoma | 2 | National Cancer Institute (NCI) | NCT04175912 | Recruiting | No Results | 52 | Study Start:  January 31, 2020  Study Completion:  October 1, 2022 | NO |
| Drug: MLN4924  Drug: Paclitaxel  Drug: Gemcitabine  Drug: Docetaxel  Drug: Carboplatin | Solid Tumours | 1 | Millennium Pharmaceuticals, Inc. \| Takeda | NCT01862328 | Completed | Not Reported | 64 | Study Start:  June 10, 2013  Study Completion:  May 21, 2018 | NO |
| Drug: Azacitidine  Drug: MLN4924  Drug: Venetoclax | Acute Myeloid Leukaemia  Atypical Chronic Myeloid Leukaemia, BCR-ABL1-Negative  Chronic Eosinophilic Leukaemia, Not Otherwise Specified  Chronic Myelomonocytic Leukaemia  Myeloid Neoplasms  Myeloproliferative Neoplasms  Myeloproliferative Neoplasms, Unclassifiable  Chronic Neutrophilic Leukaemia | 1/2 | M.D. Anderson Cancer Center \| National Cancer Institute (NCI) | NCT03862157 | Recruiting | No Results | 40 | Study Start:  February 27, 2019  Study Completion:  January 1, 2024 | NO |
| Drug: MLN4924  Drug: Vincristine  Drug: Dexamethasone  Drug: PEG-asparaginase  Drug: Doxorubicin  Drug: Cytarabine  Drug: Methotrexate  Drug: Hydrocortisone | Refractory Acute Lymphoblastic Leukaemia  Relapsed Acute Lymphoblastic Leukaemia | 1 | Julio Barredo \| Takeda \| University of Miami | NCT03349281 | Recruiting | No Results | 18 | Study Start:  March 25, 2019  Study Completion:  March 2025 | NO |
| Drug: Azacitidine  Drug: MLN4924 | Chronic Myelomonocytic Leukaemia  Acute Myeloid Leukaemia | 3 | Millennium Pharmaceuticals, Inc. \| Takeda | NCT03268954 | Active | No Results | 454 | Study Start:  November 28, 2017  Study Completion:  February 28, 2025 | NO |
| Drug: MLN4924  Drug: Docetaxel  Drug: Carboplatin  Drug: Paclitaxel | Advanced Solid Neoplasms | 1 | Millennium Pharmaceuticals, Inc. \| Takeda | NCT03330106 | Active | No Results | 44 | Study Start:  November 15, 2017  Study Completion:  February 26, 2021 | NO |
| Drug: Azacitidine  Drug: MLN4924 | Chronic Myelomonocytic Leukaemia  Acute Myeloid Leukaemia | 2 | Millennium Pharmaceuticals, Inc. \| Takeda | NCT02610777 | Active | Not Reported | 120 | Study Start:  April 14, 2016  Study Completion:  January 1, 2022 | NO |
| Drug: Azacitidine  Drug: Venetoclax  Drug: MLN4924 | Acute Myelogenous Leukaemia | 2 | Ehab L Atallah \| Medical College of Wisconsin | NCT04172844 | Recruiting | No Results | 24 | Study Start:  January 13, 2020  Study Completion:  December 1, 2025 | NO |
| Drug: Azacitidine  Drug: MLN4924 | Chronic Myelomonocytic Leukaemia  Acute Myeloid Leukaemia | 3 | Millennium Pharmaceuticals, Inc. \| Takeda | NCT03268954 | Active | Not Results | 454 | Study Start:  November 28, 2017  Study Completion:  February 28, 2025 | NO |
| Drug: MLN4924  Drug: Venetoclax  Drug: Azacitidine | Acute Myeloid Leukaemia | 2 | Takeda | NCT04266795 | Recruiting | No Results | 150 | Study Start:  October 13, 2020  Study Completion:  March 18, 2024 | NO |
| Drug: Ibrutinib  Drug: MLN4924 | B-Cell Prolymphocytic Leukaemia  Recurrent Chronic Lymphocytic Leukaemia  Recurrent Diffuse Large B-Cell Lymphoma  Recurrent Follicular Lymphoma  Recurrent Lymphoplasmacytic Lymphoma  Recurrent Mantle Cell Lymphoma  Recurrent Marginal Zone Lymphoma  Recurrent Non-Hodgkin Lymphoma  Recurrent Small Lymphocytic Lymphoma  Refractory Chronic Lymphocytic Leukaemia  Refractory Diffuse Large B-Cell Lymphoma  Refractory Follicular Lymphoma  Refractory Lymphoplasmacytic Lymphoma  Refractory Mantle Cell Lymphoma  Refractory Marginal Zone Lymphoma  Refractory Non-Hodgkin Lymphoma  Refractory Small Lymphocytic Lymphoma | 1 | City of Hope Medical Center \| National Cancer Institute (NCI) | NCT03479268 | Recruiting | No Results | 30 | Study Start:  March 22, 2018  Study Completion:  April 2, 2024 | NO |
| Drug: MLN4924  Drug: Azacitidine | Acute Myeloid Leukaemia | 2 | Milton S. Hershey Medical Center \| Millennium Pharmaceuticals, Inc. \| Takeda | NCT03709576 | Terminated | Not Reported | 3 | Study Start:  July 18, 2018  Study Completion:  July 22, 2019 | NO |
| Drug: Irinotecan  Drug: Irinotecan Hydrochloride  Drug: MLN4924  Drug: Temozolomide | Recurrent Lymphoma  Recurrent Malignant Solid Neoplasms  Recurrent Primary Central Nervous System Neoplasms  Refractory Lymphoma  Refractory Malignant Solid Neoplasms  Refractory Primary Central Nervous System Neoplasms | 1 | Children´s Oncology Group \| National Cancer Institute (NCI) | NCT03323034 | Recruiting | No Results | 56 | Study Start:  November 13, 2017  Study Completion:  December 1, 2021 | NO |
| Drug: MLN4924  Drug: Docetaxel | Non-Small-Cell Lung Cancer | 2 | University of Michigan Rogel Cancer Center | NCT03228186 | Recruiting | No Results | 37 | Study Start:  March 5, 2018  Study Completion:  March 1, 2021 | NO |
| Drug: Azacitidine  Drug: Cytarabine  Drug: Fludarabine Phosphate  Drug: Methotrexate  Drug: MLN4924  Drug: Therapeutic Hydrocortisone | Acute Myeloid Leukaemia Arising From Previous Myelodysplastic Syndrome  Recurrent Acute Myeloid Leukaemia  Recurrent Myelodysplastic Syndrome  Refractory Acute Myeloid Leukaemia  Refractory Myelodysplastic Syndrome | 1 | Children's Oncology Group | NCT03813147 | Suspended | No Results | 38 | Study Start:  May 1, 2019  Study Completion:  March 31, 2021 | NO |
| Drug: MLN4924  Drug: Rifampin  Drug: Docetaxel  Drug: Carboplatin  Drug: Paclitaxel | Advanced Solid Neoplasms | 1 | Millennium Pharmaceuticals, Inc. | NCT03486314 | Active | No Results | 20 | Study Start:  August 13, 2018  Study Completion:  February 28, 2021 | NO |
| Drug: MLN4924  Drug: Pemetrexed and cisplatin | Mesothelioma | 1/2 | Memorial Sloan Kettering Cancer Center \| M.D. Anderson Cancer Center | NCT03319537 | Recruiting | No Results | 63 | Study Start:  October 5, 2017  Study Completion:  October 2022 | NO |
| Drug: Azacitidine Subcutaneous Injection or Intravenous Infusion  Drug: MLN4924 Infusion | Myeloproliferative Neoplasms | 2 | Vanderbilt-Ingram Cancer Center \| National Cancer Institute (NCI) | NCT03238248 | Recruiting | No Results | 71 | Study Start:  August 7, 2017  Study Completion:  July 2023 | NO |
| Drug: MLN4924  Drug: Fluconazole  Drug: Itraconazole  Drug: Docetaxel  Drug: Carboplatin  Drug: Paclitaxel | Advanced Solid Tumours | 1 | Millennium Pharmaceuticals, Inc. \| Takeda | NCT02122770 | Completed | Not Reported | 51 | Study Start:  April 1, 2014  Study Completion:  June 5, 2017 | NO |
| Drug: MLN4924  Drug: Azacitidine | Acute Myeloid Leukaemia | 3 | PETHEMA Foundation \| Millennium Pharmaceuticals, Inc. \| Dynamic Science S.L. | NCT04090736 | Recruiting | No Results | 466 | Study Start:  August 13, 2019  Study Completion:  June 30, 2023 | NO |
| Drug: Azacitidine  Drug: MLN4924 | Recurrent Acute Myeloid Leukaemia  Refractory Acute Myeloid Leukaemia | 2 | National Cancer Institute (NCI) | NCT03745352 | Suspended | No Results | 72 | Study Start:  May 20, 2019  Study Completion:  June 1, 2021 | NO |
| Drug: Cytarabine  Drug: Idarubicin  Drug: MLN4924 | Acute Myeloid Leukaemia Arising From Previous Myelodysplastic Syndrome  Acute Myeloid Leukaemia With Myelodysplasia-Related Changes  Therapy-Related Acute Myeloid Leukaemia | 1/2 | University of Southern California \| National Cancer Institute (NCI) | NCT03330821 | Recruiting | No Results | 53 | Study Start:  April 18, 2018  Study Completion:  April 18, 2021 | NO |
| Drug: MLN4924 | Metastatic Melanoma | 1 | Millennium Pharmaceuticals, Inc. | NCT01011530 | Completed | Partial Response; Stable Disease | 37 | Study Start:  December 2009  Study Completion:  January 2013 | (106) |
| Drug: Carboplatin  Drug: Paclitaxel  Drug: MLN4924 | Metastatic Lung Non-Small-Cell Squamous Carcinoma  Metastatic Lung Non-Squamous Non-Small-Cell Carcinoma  Stage IIIB Lung Cancer AJCC v8  Stage IV Lung Cancer AJCC v8  Stage IVA Lung Cancer AJCC v8  Stage IVB Lung Cancer AJCC v8  Unresectable Lung Non-Small Cell Carcinoma  Unresectable Lung Non-Squamous Non-Small Cell Carcinoma | 2 | National Cancer Institute (NCI) | NCT03965689 | Recruiting | No Results | 25 | Study Start:  September 3, 2019  Study Completion:  February 26, 2021 | NO |
| Drug: Azacitidine  Drug: MLN4924  Drug: Docetaxel  Drug: Paclitaxel  Drug: Carboplatin | Neoplasms  Acute Myeloid Leukaemia  Chronic Myelomonocytic Leukaemia | 1 | Millennium Pharmaceuticals, Inc. \| Takeda | NCT03814005 | Recruiting | No Results | 42 | Study Start:  July 10, 2019  Study Completion:  February 22, 2022 | NO |
| Drug: MLN4924  Drug: Azacitidine | Acute Myelogenous  Leukemia | 1 | Millennium Pharmaceuticals, Inc.\| Takeda | NCT01814826 | Completed | Complete Response; Partial Response | 64 | Study Start:  April 10, 2013  Study Completion:  April 8, 2018 | (144) |
